# Learning curve for endoscopic retrograde appendicitis therapy: a multicenter retrospective study with different levels of trainees

**DOI:** 10.1093/gastro/goag053

**Published:** 2026-05-29

**Authors:** Jiyu Zhang, Jianping Fan, Haipeng Yuan, Miao Shi, Saif Ullah, Antonio Marin Garcia, Mohammad Serajul Islam, Jianfei Feng, Ning Su, Haiyang Li, Bingrong Liu, Dan Liu

**Affiliations:** Department of Gastroenterology and Hepatology, The First Affiliated Hospital of Zhengzhou University, Zhengzhou, Henan 450052, P. R. China; Endoscopy Center, Jingxing County Hospital, Shijiazhuang, Hebei 050300, P. R. China; Department of Gastroenterology,The Affiliated Taian City Central Hospital of Qingdao University , Taian, Shandong 271000, P. R. China; Department of Gastroenterology and Hepatology, The First Affiliated Hospital of Zhengzhou University, Zhengzhou, Henan 450052, P. R. China; Department of Gastroenterology and Hepatology, The First Affiliated Hospital of Zhengzhou University, Zhengzhou, Henan 450052, P. R. China; Gastroenterology Department, Vall d‘Hebron University Hospital, Barcelona, Spain; Department of Gastroenterology and Hepatology, The First Affiliated Hospital of Zhengzhou University, Zhengzhou, Henan 450052, P. R. China; Endoscopy Center, Jingxing County Hospital, Shijiazhuang, Hebei 050300, P. R. China; Department of Gastroenterology and Hepatology, The First Affiliated Hospital of Zhengzhou University, Zhengzhou, Henan 450052, P. R. China; Department of Gastroenterology and Hepatology, The First Affiliated Hospital of Zhengzhou University, Zhengzhou, Henan 450052, P. R. China; Department of Gastroenterology and Hepatology, The First Affiliated Hospital of Zhengzhou University, Zhengzhou, Henan 450052, P. R. China; Henan Key Medical Laboratory: Innovative Technology for Minimally Invasive Treatment of Digestive Endoscope, The First Affiliated Hospital of Zhengzhou University, Zhengzhou, Henan 450001, P. R. China; Department of Gastroenterology and Hepatology, The First Affiliated Hospital of Zhengzhou University, Zhengzhou, Henan 450052, P. R. China

**Keywords:** endoscopic retrograde appendicitis therapy, acute appendicitis, learning curve, endoscopic technique, endoscopic retrograde cholangiopancreatography

## Abstract

**Background and aim:**

Endoscopic retrograde appendicitis therapy (ERAT) is a minimally invasive treatment for appendicitis, widely practiced in China. Clarifying the learning curve, especially among endoscopists with varying endoscopic experience, including endoscopic retrograde cholangiopancreatography (ERCP) skills, is essential for its broader adoption. This study aimed to determine the number of cases required to achieve procedural proficiency across different experience levels, as well as to examine the disparities in technical success rates and clinical outcomes.

**Methods:**

In this multicenter retrospective study, 655 consecutive patients who had undergone ERAT between October 2021 and March 2024 in The First Affiliated Hospital of Zhengzhou University, Jingxing County Hospital, and The Affiliated Taian City Central Hospital of Qingdao University were analyzed. All ERAT procedures were performed by nine trainees from three hospitals, categorized into novice, non-ERCP experience, and ERCP experience groups. Outcomes included technical/clinical success, procedure time, pain, recurrence, length of stay, and adverse events. Learning curves were evaluated with cumulative sum (CUSUM) based on procedure time.

**Results:**

Technical and clinical outcomes improved with increasing experience in all groups. Proficiency occurred at the 31st case in the ERCP group, the 55th in the non‑ERCP group, and the 59th in novices. The ERCP group had the highest technical (99.1%) and clinical (94.4%) success (*P *= 0.023 and *P *= 0.093), the shortest mean procedure time (20.9 minutes; *P *< 0.001), and the lowest recurrence (*P *< 0.001). Complication rates, including intraoperative and postoperative adverse events, were low and similar across groups (0.8%–2.6%, *P *> 0.05). Postoperative pain and hospital stay decreased with experience (*P *< 0.05).

**Conclusions:**

Endoscopists with extensive prior endoscopic experience, particularly in ERCP, achieve proficiency in ERAT more rapidly. Structured training programs tailored to different experience levels are essential to support the safe and effective global implementation of ERAT.

## Introduction

Appendicitis is one of the most common acute abdominal conditions. In most cases, appendicitis arises due to obstruction of the appendiceal lumen by various factors, such as fecaliths, parasites, or hypertrophic lymphoid tissue, which leads to bacterial overgrowth, inflammation of the appendiceal wall, and increased intraluminal pressure [1]. In severe cases, ischemia of the appendiceal wall may occur, resulting in gangrene and perforation. Appendectomy and conservative antibiotic therapy have long been the only treatment options for appendicitis. Through extended clinical practice and contemplation, inspired by endoscopic retrograde cholangiopancreatography (ERCP), Liu *et al.* [2] proposed a novel endoscopic approach for the treatment of appendicitis named endoscopic retrograde appendicitis therapy (ERAT). The procedure involves the use of a colonoscope to perform appendiceal catheterization, decompression, irrigation, removal of appendicoliths, and stenting [3]. Since its introduction in 2012, ERAT has been predominantly adopted and performed in China, where it has gained widespread recognition [4–7]. Additionally, this technique has attracted considerable interest among endoscopists in other countries, highlighting its potential for global dissemination [8, 9]. Given the growing interest in ERAT, it is crucial to define the learning curve for learners with varying levels of experience in therapeutic endoscopy, especially distinguishing between endoscopists with prior experience in ERCP and those without such experience. Since ERAT and ERCP share substantial procedural similarities, it is crucial to determine whether physicians with ERCP experience can master the ERAT technique more rapidly and proficiently. This has major implications for the international promotion of ERAT and may encourage more endoscopists with ERCP experience to learn this novel minimally invasive diagnostic and therapeutic technique in digestive endoscopy.

As one of the leading endoscopy centers where ERAT originated, our hospital has provided extensive training for trainees nationwide through lectures, demonstrations, and hands-on workshops. We have accepted many trainees with different baseline levels of endoscopic experience, including experience in ERCP. In this study, we aimed to evaluate the learning process and the number of cases required to achieve ERAT proficiency for novice, non-ERCP experience, and ERCP experience groups. Furthermore, we explores the differences in technical success rates and clinical outcomes among the various groups.

## Methods

### Study design and population

We performed a multicenter retrospective analysis of prospectively collected data from The First Affiliated Hospital of Zhengzhou University, Jingxing County Hospital, and The Affiliated Taian City Central Hospital of Qingdao University between October 2021 and March 2024.

Inclusion criteria were as follows: (i) patients aged between 18 and 60 years; (ii) patients diagnosed with suspected (or not excluded) acute appendicitis by abdominal computed tomography (CT) or ultrasound; and (iii) patients who received ERAT treatment. Exclusion criteria included as follows: (i) patients found to have complicated appendicitis based on preoperative examination or intraoperative findings; or (ii) patients lost to follow-up after procedure. Finally, the study included 655 consecutive patients with acute appendicitis (338 males, with a median age of 32 years [interquartile range (IQR): 23–48 years] who underwent ERAT performed by nine trainees [five males, with a median age of 42 years [IQR: 38–45 years]). All trainees have graduated from the structured ERAT training program led by Dr Bingrong Liu, the creator of the ERAT technique. The training program included systematic theoretical instruction as well as observation and study of no fewer than 10 ERAT procedures. Based on the background of endoscopic experience, the trainees were divided into three groups, namely the novice group, with endoscopic experience limited to upper and colonoscopic endoscopy (each trainee has less than 1 year of experience with conventional endoscopy, and the number of procedures performed does not exceed 1,000 cases); the non-ERCP experience group, with experience in therapeutic endoscopy, but not in ERCP (each trainee has no less than 5 years of experience in therapeutic endoscopy, including procedures such as endoscopic mucosal resection and endoscopic submucosal dissection); and the ERCP experience group, with experience in therapeutic endoscopy, which includes more than 2 years of ERCP experience, having completed over 100 ERCP procedures. The basic characteristics of the trainees in each group, as well as the number of patients treated by each trainee (Table 1).

**Table 1:** Basic characteristics of the trainees and the number of patients treated by each trainee

Variable	Group	Sex	Age	Number of patients
Trainee 1	Novice	Male	35 years	92
Trainee 2	Novice	Female	40 years	81
Trainee 3	Novice	Male	42 years	73
Trainee 4	Non-ERCP expert	Male	45 years	45
Trainee 5	Non-ERCP expert	Female	42 years	64
Trainee 6	Non-ERCP expert	Female	57 years	86
Trainee 7	ERCP expert	Female	35 years	75
Trainee 8	ERCP expert	Male	61 years	64
Trainee 9	ERCP expert	Male	38 years	75

ERCP, endoscopic retrograde cholangiopancreatography.

Data on patient demographic characteristics, preoperative characteristics, procedural details, and postoperative outcomes were collected. The diagnosis of acute appendicitis was established based on medical history, physical examination, and abdominal imaging findings. Procedure time was defined as the duration from appendiceal catheterization to the completion of intra-appendiceal lumen manipulation, including stenting. Technical success was defined as successful appendiceal intubation, irrigation, appendicolith removal, and stenting if needed. In addition, clinical success was defined as symptom relief, which was maintained for at least 1 month. Other outcomes included postoperative visual analog scale (VAS) pain scores, laboratory findings, conversion to surgery, length of hospital stay, and recurrence. Recurrent appendicitis was defined as recurrent symptoms 1 month following ERAT, which was confirmed by abdominal CT or ultrasound examination, characterized by an appendiceal diameter > 6 mm, cecal wall thickening, periappendiceal edema, and/or small amounts of effusion.

### ERAT procedure

**Figure 1:**
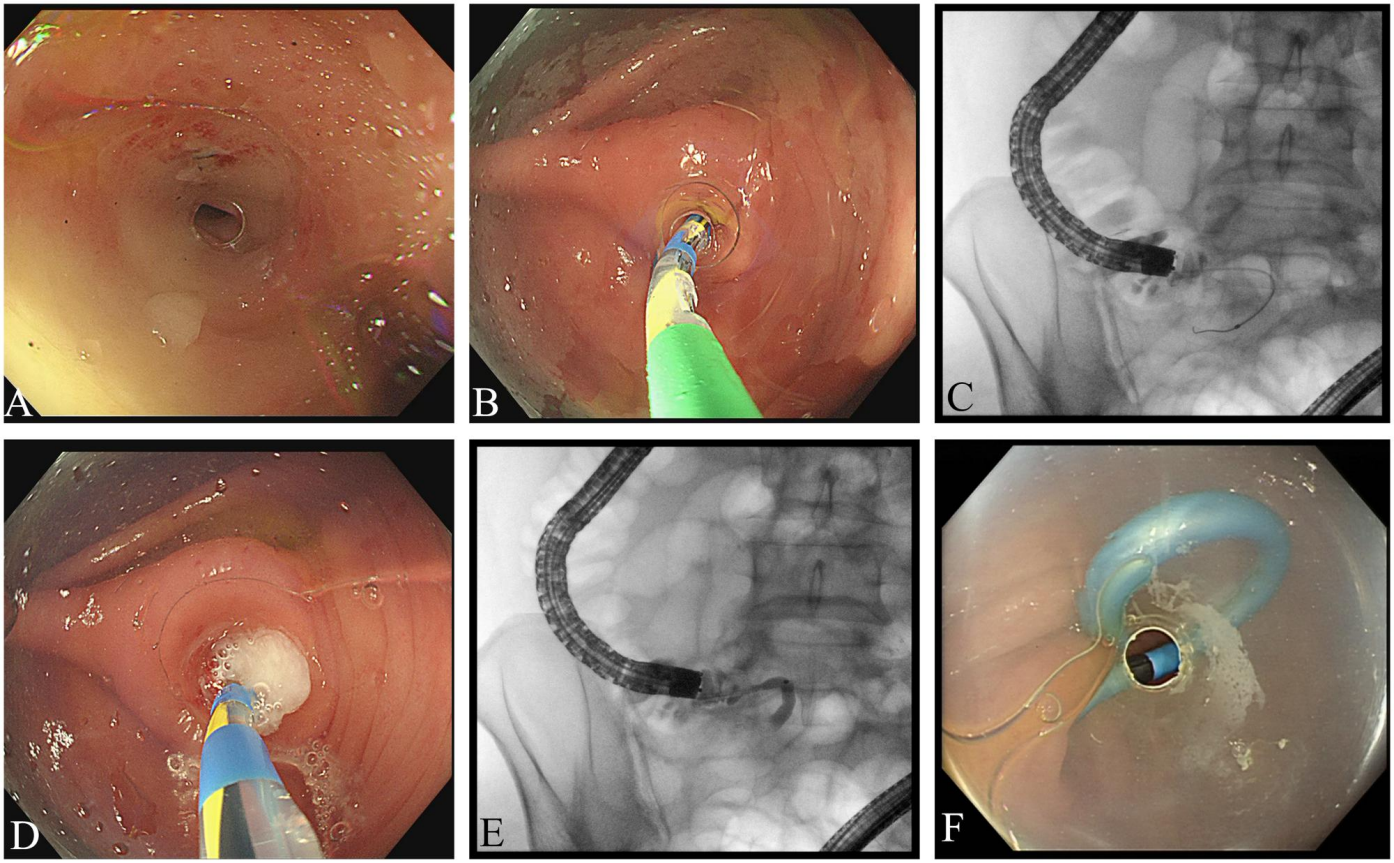
The procedure of endoscopic retrograde appendicitis therapy. (A) Identification of the appendiceal orifice. (B) and (C) Appendiceal intubation using guidewire and catheter techniques under X-ray guidance. (D) Irrigation of the appendiceal lumen. (E) Appendiceal contrast imaging. (F) Appendiceal stent placement.

The bowel preparation for ERAT is the same as that for routine colonoscopy, typically utilizing oral bowel-cleansing agents such as polyethylene glycol. For patients requiring emergency ERAT, a cleansing enema can be considered. Following adequate bowel preparation, a colonoscope (OLYMPUS CF-H290L, Tokyo, Japan) was advanced to the cecum, and the appendiceal orifice was carefully examined for possible signs of appendicitis, such as congestion, edema, or suppuration. A tapered transparent cap (Henan DIYI, Zhengzhou, P. R. China) was utilized to facilitate appendiceal intubation. After successful appendiceal cannulation, a diluted contrast agent (Iohexol Injection) was injected to demonstrate the morphology of the appendix and identify signs of filling defects. Decompression of the appendiceal lumen was then performed by aspiration via catheter, and saline irrigation was used to expel pus and fecaliths (Fig. 1).

When the presence of fecaliths was confirmed, endoscopic extraction basket or retrieval balloon was employed under radiographic guidance. Laser lithotripsy under digital single-operator cholangioscope (eyeMax; Micro-Tech, Nanjing, P. R. China) was used as a remedial method if the fecalith was too large to be removed by the extraction basket. A 7–8.5-Fr plastic stent (5–7 cm in length) was placed if required. The stent was typically scheduled for removal during an outpatient visit approximately 1 month later, although spontaneous expulsion of the stent from the appendiceal lumen could also occur.

### Cumulative sum analysis

Cumulative sum (CUSUM) analysis, originally introduced by de Leval *et al.* [10] to assess surgical performance, was used to quantitatively evaluate the learning curve for ERAT. CUSUM was defined as the CUSUM of the differences between individual data points and a predefined target value. In this study, the target value was the mean procedure time across all cases in each group. All cases performed by the endoscopists were sequentially numbered in the order of their acceptance for ERAT. CUSUM for the first case was calculated as the difference between the average procedure time of case 1 for each of the three trainees in the group and the average procedure time for all ERAT procedures within that group. CUSUM for the second case was derived by adding the previous CUSUM value to the difference between each trainee’s average procedure time for their second patient and the overall average procedure time for all patients. This process was repeated until the final case’s CUSUM was calculated, with the series ultimately summing to zero. Transition points marking completion of the learning phase were identified as inflection points on the CUSUM graph, defined as the point where the gradient turned negative for three or more consecutive cases. Due to a low incidence of complications, risk-adjusted CUSUM and success/failure charts were not generated.

### Statistical analysis

Categorical variables were expressed as counts (percentages) and compared using the chi-square or Fisher’s exact test as appropriate. Cochran–Armitage trend tests were used for ordered groups. Continuous variables were presented as means ± standard deviations and compared using one-way ANOVA with Welch’s correction. Non-normally distributed or ordinal variables were expressed as medians (IQR) and analyzed using the Kruskal–Wallis test with Bonferroni adjustment. Binary outcomes over time were evaluated with McNemar’s or trend tests. Survival outcomes, such as time to recurrence, were compared by Kaplan–Meier analysis with log-rank test. Associations between variables and outcomes were expressed as odds ratios (ORs) or hazard ratios (HRs) with 95% confidence intervals (CIs). All analyses were performed using SPSS version 28 (IBM Corp., USA) and R version 4.3.0 (R Foundation). A two-sided *P *< 0.05 was considered significant.

### Ethics statement

This study was conducted in accordance with the ethical standards of the Declaration of Helsinki and was approved by the Institutional Review Board of the First Affiliated Hospital of Zhengzhou University (approval No. 2023-KY-0755), with the ethical document also accepted by the other two participating institutions. Informed consent was waived due to the retrospective nature of the study.

## Results

In this study, a total of 655 patients underwent ERAT performed by nine trainees in three endoscopic centers, namely 246 cases by novice endoscopists, 195 cases by non-ERCP experience endoscopists, and 214 cases by ERCP experience endoscopists. Male gender distribution in these three groups of patients was 55.7%, 47.7%, and 50.5%, respectively (*P *= 0.221), and average age was 33.1 ± 18.9, 32.9 ± 19.7, and 30.1 ± 19.3 years, respectively (*P *= 0.297). Baseline characteristics, including body mass index, preoperative VAS scores, fever, laboratory results, and fecalith appendicitis, showed no significant differences among the groups. However, appendiceal stent placement rate was significantly higher in both expert groups compared with the novice group (*P *< 0.001) (Table 2).

**Table 2:** Demographic characteristics, clinical characteristics, procedural details, and outcomes for cases undergoing ERAT in three groups

Variable	Novice group (*n *= 246)	Non-ERCP experience group (*n *= 195)	ERCP experience group (*n *= 214)	*P*-value
Male, *n* (%)	137 (55.7)	93 (47.7)	108 (50.5)	0.221
Age, years, mean ± SD	33.1 ± 18.9	32.9 ± 19.7	30.1 ± 19.3	0.297
BMI, kg/m^2^, *n* (%)				0.185
Underweight (BMI < 18.5 kg/m^2^)	30 (12.2)	22 (11.3)	28 (13.1)	
Normal weight (18.5 ≤ BMI <25 kg/m^2^)	144 (58.5)	118 (60.5)	116 (54.2)	
Overweight (25 ≤ BMI <30 kg/m^2^)	62 (25.2)	40 (20.5)	58 (27.1)	
Obesity (BMI ≥ 30 kg/m^2^)	10 (4.1)	15 (7.7)	12 (5.6)	
Preoperative VAS score, median (IQR)	7 (4–7)	7 (4–7)	7 (4–7)	0.872
Preoperative VAS score, *n* (%)				0.981
Mild pain (1–3)	21 (8.5)	17 (8.7)	16 (7.5)	
Moderate pain (4–6)	86 (35.0)	69 (35.4)	73 (34.1)	
Severe pain (7–10)	139 (56.5)	109 (55.9)	125 (58.4)	
Fever, *n* (%)	21 (8.5)	21 (10.8)	18 (8.4)	0.601
Preoperative laboratory tests, *n* (%)				
WBC > 10.0 × 10^9^/L	68 (27.6)	41 (21.0)	56 (26.2)	0.261
Neutrophils > 70%	84 (34.1)	64 (32.8)	83 (38.8)	0.405
CRP > 5 mg/L	85 (34.6)	69 (35.4)	92 (43.0)	0.133
Fecalith appendicitis, *n* (%)	130 (52.8)	98 (50.3)	102 (47.7)	0.519
Procedure time, min, mean ± SD	31.4 ± 9.1	27.0 ± 7.3	20.9 ± 5.2	<0.001
Appendiceal stent placement, *n* (%)	20 (8.1)	81 (41.5)	91 (42.5)	<0.001
Technical success rate, *n* (%)	233 (94.7)	183 (93.8)	212 (99.1)	0.023
Conversion to surgery, *n* (%)	10 (4.1)	4 (2.1)	4 (1.9)	0.307
Intraoperative complications, *n* (%)	4 (1.6)	5 (2.6)	3 (1.4)	0.669
Perforation	4 (1.6)	3 (1.5)	2 (0.9)	
Hemorrhage	3 (1.2)	3 (1.5)	2 (0.9)	
Delayed perforation, *n* (%)	2 (0.8)	2 (1.0)	0 (0.0)	0.550
Postoperative VAS score at 6 h, median (IQR)	1 (0–2)	1 (0–2)	0 (0–2)	< 0.001
Postoperative VAS score at 6 h, *n* (%)				< 0.001
No pain (0)	91 (37.0)	93 (47.7)	115 (53.7)	
Mild pain (1–3)	142 (57.7)	91 (46.7)	93 (43.5)	
Moderate pain (4–6)	13 (5.3)	11 (5.6)	6 (2.8)	
Severe pain (7–10)	0 (0)	0 (0)	0 (0)	
Postoperative length of stay, days, mean (SD)	1.8 ± 1.7	1.8 ± 1.6	1.9 ± 1.0	0.726
Postoperative laboratory tests,^a^ *n* (%)	112	152	170	
WBC > 10.0 × 10^9^/L	14 (12.5)	21 (13.8)	25 (14.7)	0.871
Neutrophils > 70%	19 (17.0)	32 (21.0)	37 (21.8)	0.592
CRP > 5 mg/L	14 (12.5)	27 (17.8)	29 (17.1)	0.472
Recurrent appendicitis, *n* (%)	51 (20.7)	22 (11.3)	18 (8.4)	< 0.001
Time to recurrence, months, median (IQR)	2.00 (1.00–6.00)	9.00 (4.50–16.50)	17.00 (10.00–21.25)	< 0.001
Additional operation, *n* (%)	51	22	18	< 0.001
Appendectomy	29 (56.9)	8 (36.4)	3 (16.7)	
Repeated ERAT	5 (9.8)	5 (22.7)	7 (38.9)	
Conservative treatment	17 (33.3)	9 (40.9)	8 (44.4)	
Clinical success rate, *n* (%)	213 (86.6)	178 (91.3)	197 (92.1)	0.093

a Since not all patients underwent laboratory tests postoperatively, only a subset of available results was collected; the number of cases who received postoperative laboratory tests in novice group, non-ERCP experience group, and ERCP experience group are 112, 152, and 170, respectively.

ERAT, endoscopic retrograde appendicitis therapy; ERCP, endoscopic retrograde cholangiopancreatography; BMI, body mass index; VAS, visual analog scale; IQR, interquartile range; SD, standard deviation; WBC, white blood cell count; CRP, C-reactive protein.

For the technical success rate, the ERCP experience group had a significantly higher rate than both the non-ERCP experience group and the novice group (99.1% vs 93.8%, *P *= 0.005; 99.1% vs 94.7%, *P *= 0.008). Conversion to surgery (1.9%–4.1%), intraoperative complications (perforation, hemorrhage) (1.4%–2.6%), and postoperative complications (e.g. delayed perforation) (0–0.8%) were low across all groups, without significant inter-group differences. For postoperative VAS score at 6 h, pairwise comparison showed that the ERCP experience group had a significantly better postoperative pain score than the novice group (*P *= 0.001), whereas the difference between the non-ERCP experience group and the novice group did not reach statistical significance (*P *= 0.063). Postoperative hospital stays were similar (1.8 ± 1.7, 1.8 ± 1.6, and 1.9 ± 1.0 days; *P *= 0.726). Postoperative laboratory parameters improved relative to preoperative values in all groups, but with no significant differences (both *P > *0.05) (Table 2).

**Figure 2:**
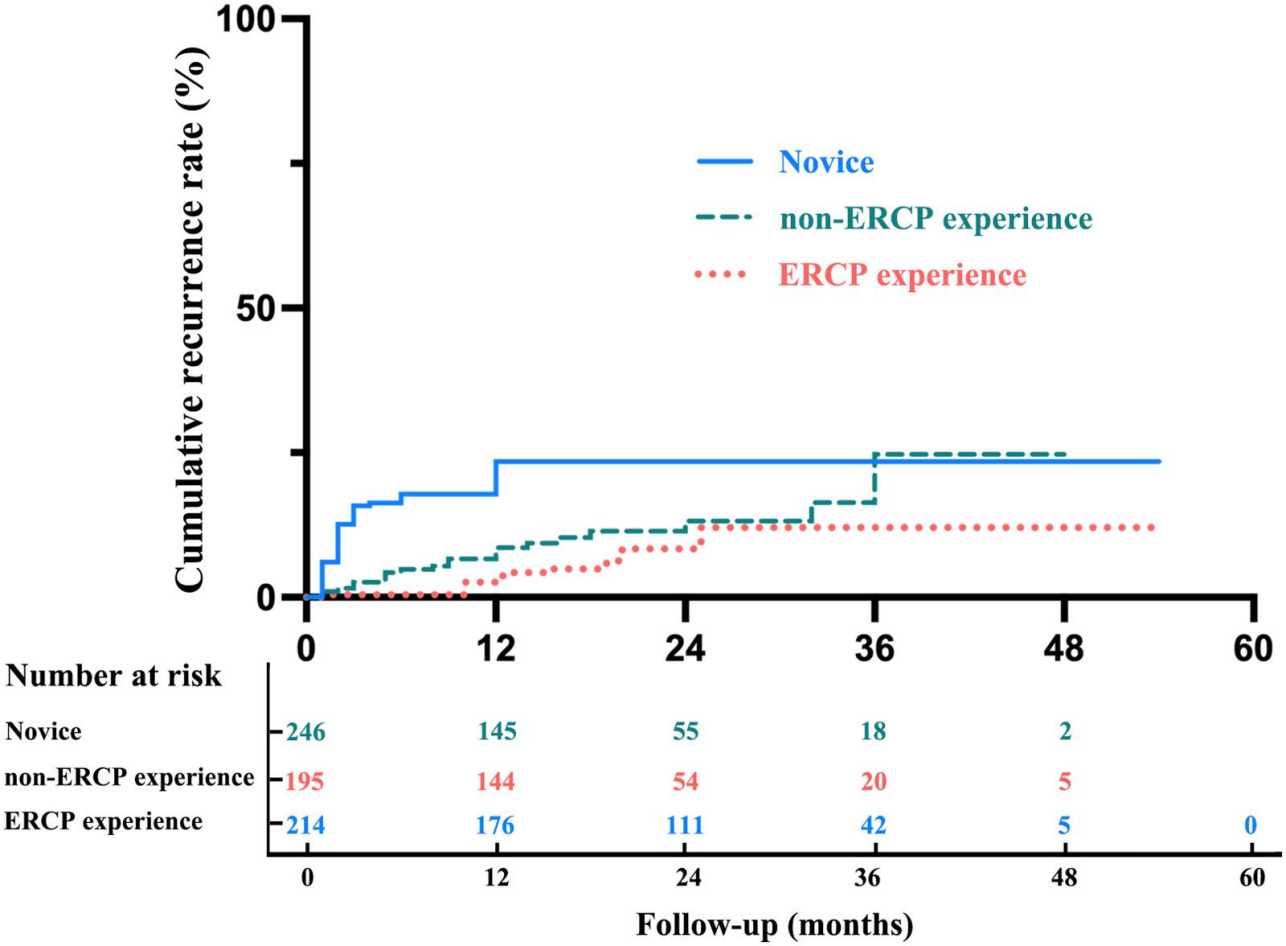
Comparison of cumulative recurrence rates among the novice, non-ERCP experience, and ERCP experience groups. ERCP, endoscopic retrograde cholangiopancreatography.

Clinical success rate was higher in the experience groups than in the novice group, though difference between the two experience groups was not significant (*P *= 0.093). For recurrent appendicitis during follow-up, the recurrence rate in the novice group was significantly higher than those in both the non-ERCP experience group and the ERCP experience group (20.7% vs 11.3%, *P *= 0.012; 20.7% vs 8.4%, *P *< 0.001). The median recurrence time was 5 (2–12) months after ERAT. Of these with recurrence, 34 patients received conservative therapy, 17 repeated ERAT, and 40 appendectomy. The median recurrence time was shorter in the novice group [2.00 (1.00–6.00) months] compared with the expert groups [17.00 (10.00–21.25) months] (*P *< 0.001). There were significant inter-group differences in additional operations for recurrence (*P *< 0.001); namely, the novice-treated patients more likely had appendectomy (56.9%), while expert-treated patients were more likely to receive repeated ERAT (38.9%) (Fig. 2).

We further compared several key characteristics between the non-ERCP experience group and ERCP experience group. It was found that the experience group had a shorter procedure time (*P *< 0.001), a higher technical success rate (*P *= 0.005), a lower recurrence rate of appendicitis (*P *= 0.418), and a shorter time to recurrence (*P *< 0.001) (Table 3). However, there was no significant difference in the stent placement rate between the two groups (*P *= 1.000). Furthermore, an analysis was conducted within each group to examine the relationship between appendiceal stent placement and recurrence. It was found that in all groups, the recurrence rate was higher among those without stent placement (*P *= 0.298, *P *= 0.057, and *P *= 0.186, respectively) (Table 4).

**Table 3:** Further comparison of the characteristics that showed differences among the three groups was conducted between the non-ERCP experience group and the ERCP experience group

Variable	Non-ERCP experience group (*n *= 195)	ERCP experience group (*n *= 214)	*P*-value
Procedure time, min, mean ± SD	27.0 ± 7.3	20.9 ± 5.2	<0.001
Appendiceal stent placement, *n* (%)	83 (42.6)	91 (42.5)	1.000
Technical success rate, *n* (%)	183 (93.8)	212 (99.1)	0.005
Postoperative VAS score at 6 h, median (IQR)	1 (0–2)	0 (0–2)	0.154
Postoperative VAS score at 6 h, *n* (%)			0.154
No pain (0)	93 (47.7)	115 (53.7)	
Mild pain (1–3)	91 (46.7)	93 (43.5)	
Moderate pain (4–6)	11 (5.6)	6 (2.8)	
Severe pain (7–10)	0 (0.0)	0 (0.0)	
Recurrent appendicitis, *n* (%)	22 (11.3)	18 (8.4)	0.418
Time to recurrence, months, median (IQR)	9.00 (4.50–16.50)	17.00 (10.00–21.25)	<0.001
Additional operation, *n* (%)	22	18	0.319
Appendectomy	8 (36.4)	3 (16.7)	
Repeated ERAT	5 (22.7)	7 (38.9)	
Conservative treatment	9 (40.9)	8 (44.4)	

ERCP, endoscopic retrograde cholangiopancreatography; SD, standard deviation; VAS, visual analog scale; IQR, interquartile range; ERAT, endoscopic retrograde appendicitis therapy.

**Table 4:** The relationship between appendiceal stent placement and recurrence in novice, non-ERCP experience, and ERCP experience groups

Variable	Appendiceal stent placement	No appendiceal stent placement	*P*-value
Novice group	*n *= 20	*n *= 226	0.298
Recurrent appendicitis, *n* (%)	2 (10.0)	44 (19.5)	
No recurrent appendicitis, *n* (%)	18 (90.0)	182 (80.5)	
Non-ERCP experience group	*n *= 81	*n *= 114	0.057
Recurrent appendicitis, *n* (%)	5 (6.2)	17 (14.9)	
No recurrent appendicitis, *n* (%)	76 (93.8)	97 (85.1)	
ERCP experience group	*n *= 91	*n *= 123	0.186
Recurrent appendicitis, *n* (%)	5 (5.5)	13 (10.6)	
No recurrent appendicitis, *n* (%)	86 (94.5)	110 (89.4)	

ERCP, endoscopic retrograde cholangiopancreatography.

**Figure 3:**
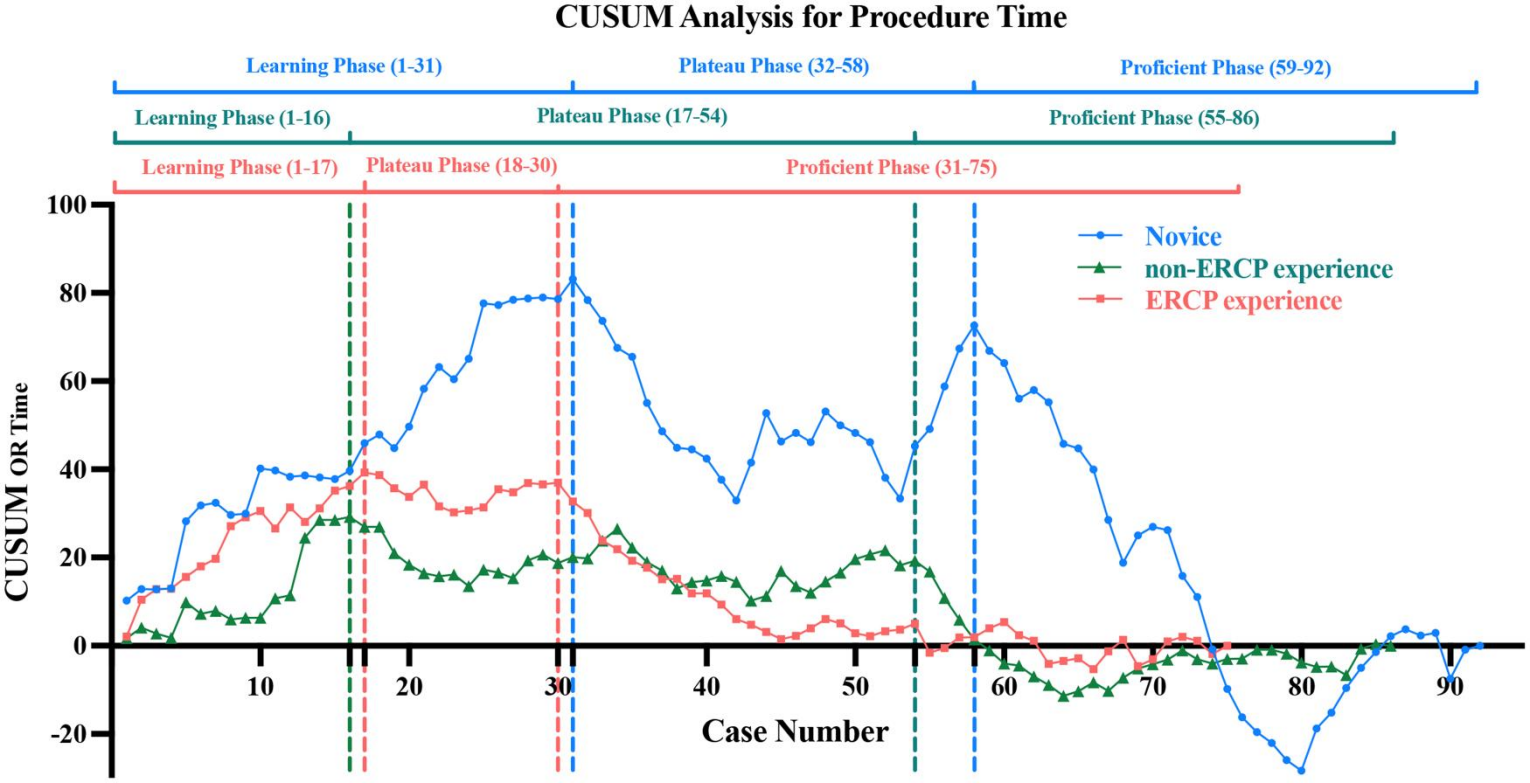
Cumulative sum (CUSUM) analysis of procedure times for the novice, non-ERCP experience, and ERCP experience groups.

The endoscopists in the novice group took 31.4 ± 9.1 minutes to complete ERAT, which was significantly longer compared with the endoscopists in the non-ERCP experience (27.0 ± 7.3 minutes, *P *< 0.001) and the ERCP experience groups (20.9 ± 5.2 minutes, *P *< 0.001). The linear trend lines for procedure time in the three groups, represented by the equations *y*1 = −0.5582*x*_1_ + 61.516 (*R*^2^ = 0.2745), *y*_2_ = −0.2746*x*_2_ + 21 (*R*^2^ = 0.3751), and *y* = −0.5013*x* + 33.844 (*R*^2^ = 0.566), showed that procedure time decreased as case volume increased. The CUSUM analysis of the procedure time for all groups identified three distinct phases of skill acquisition as shown in Fig. 3:

1. *Learning phase*: Cases 1–31 (novices), 1–16 (non-ERCP experience), 1–17 (ERCP experience). This phase reflects a high variability as endoscopists acquired basic skills and became familiar with ERAT procedure. The average procedure times were 34.1 ± 7.6 minutes, 28.8 ± 8.5 minutes, and 23.1 ± 5.8 minutes in the novice, non-ERCP experience, and ERCP experience groups, respectively.
2. *Plateau phase*: Cases 32–58 (novices), 17–54 (non-ERCP experience), 18–30 (ERCP experience). Procedure times significantly decreased to 31.0 ± 11.2 minutes, 26.7 ± 7.7 minutes, and 20.4 ± 5.4 minutes in the novice, non-ERCP experience, and ERCP experience groups, respectively, indicating the improvement of procedural efficiency and partial skill consolidation.
3. *Proficient phase*: Cases 59–92 (novices), 55–86 (non-ERCP experience), 31–75 (ERCP experience). Average procedure times stabilized at 28.4 ± 7.3 minutes, 25.6 ± 3.7 minutes, and 20.2 ± 4.5 minutes in the novice, non-ERCP experience, and ERCP experience groups, respectively, representative of sufficient proficiency.

**Figure 4:**
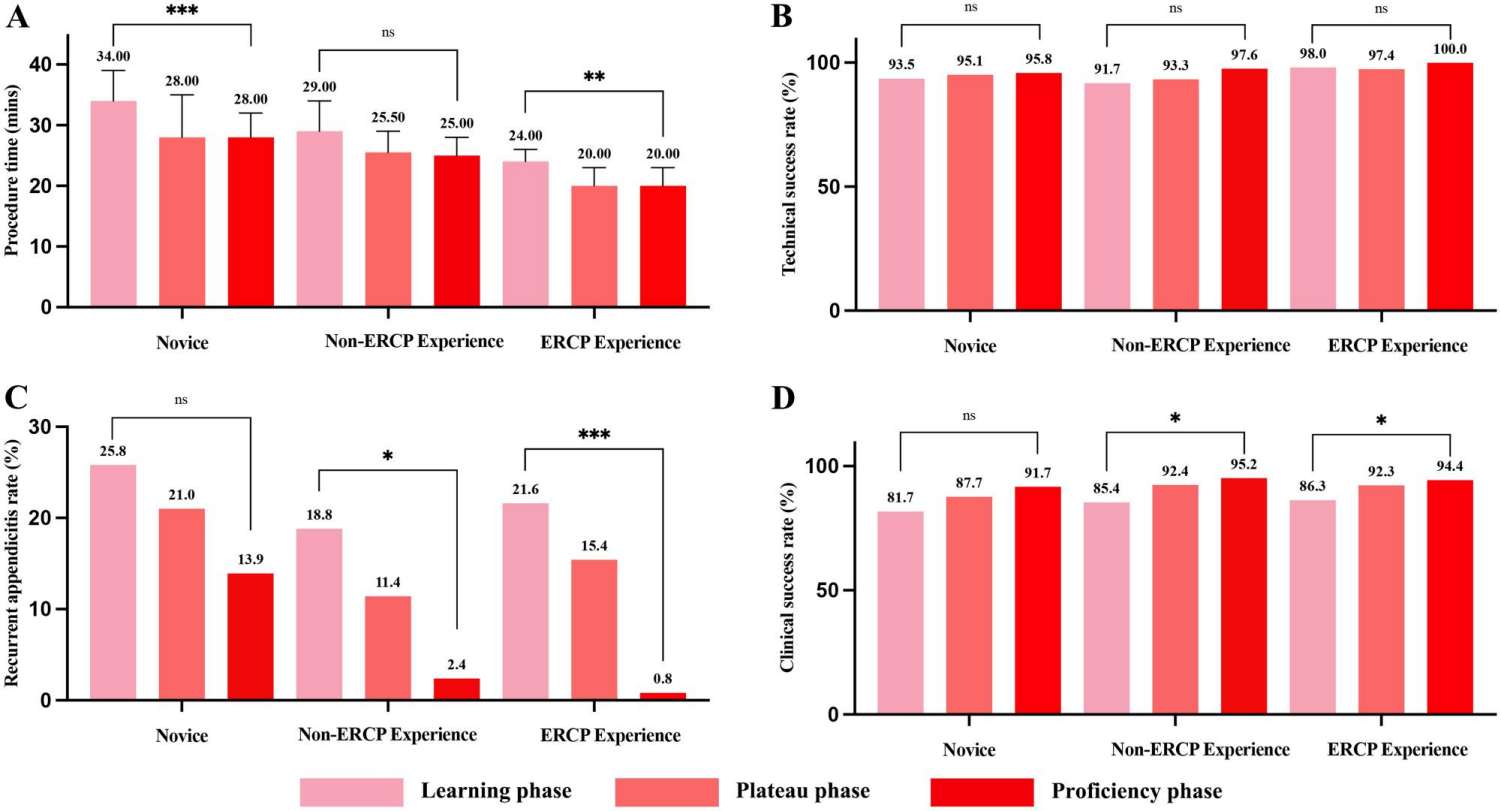
Performance metrics across learning stages. (A) Procedure time; (B) technical success rate; (C) recurrence rate; (D) clinical success rate.*P < 0.05, **P < 0.01, ***P < 0.001; ns = not significant.

The differences in procedure time among the three groups were statistically significant (Fig. 4A). Regarding the CUSUM analysis, there were no significant differences in demographic or clinical features in different learning phases. Intraoperative and postoperative complication rates also remained consistent across the three phases. Postoperative laboratory tests demonstrated a reduction in the percentage of patients with white blood cell (WBC) > 10.0 × 10^9^/L, neutrophils > 70%, and C-reactive protein (CRP) > 5 mg/L (Table 5).

**Table 5:** Comparison of patient characteristics and outcomes across the three phases of learning for three groups of trainees

Variable	Novice group				Non- ERCP experience group				ERCP experience group			
	Learning phase (Cases 1st–31st) *n *= 93	Plateau phase (Cases 32nd–58th) *n *= 81	Proficiency phase (Cases 59th–92nd) *n *= 72	*P*-value	Learning phase (Cases 1st–16th) *n *= 48	Plateau phase (Cases 17th–54th) *n *= 105	Proficiency phase (Cases 55th–86th) *n *= 42	*P-*value	Learning phase (Cases 1st–17th) *n *= 51	Plateau phase (Cases 18th–30th) *n *= 39	Proficiency phase (Cases 31st–75th) *n *= 124	*P-*value
Male, *n* (%)	52 (55.9)	42 (51.9)	43 (59.7)	0.651	21 (43.8)	51 (48.6)	21 (50.0)	0.781	27 (52.9)	19 (48.7)	62 (50.0)	0.914
Age, years, mean ± SD	34.9 ± 17.5	36.1 ± 21.2	34.3 ± 18.2	0.788	37.2 ± 19.2	34.9 ± 18.2	33.0 ± 19.4	0.412	29.1 ± 20.7	29.1 ± 21.6	30.8 ± 18.0	0.841
BMI, *n* (%)				0.057				0.228				0.136
Underweight (BMI <18.5 kg/m^2^)	9 (9.7)	16 (19.8)	6 (8.3)		7 (14.6)	9 (8.6)	5 (11.9)		7 (13.7)	7 (17.9)	26 (21.0)	
Normal weight (18.5 ≤ BMI < 25 kg/m^2^)	57 (61.3)	42 (51.9)	44 (61.1)		31 (64.6)	66 (62.9)	24 (57.1)		22 (43.1)	18 (46.2)	61 (49.2)	
Overweight (25 ≤ BMI <30 kg/m^2^)	23 (24.7)	19 (23.5)	20 (27.8)		5 (10.4)	23 (21.9)	12 (28.6)		20 (39.2)	10 (25.6)	31 (25.0)	
Obesity (BMI ≥ 30 kg/m^2^)	4 (4.3)	4 (4.9)	2 (2.8)		3 (6.3)	8 (7.6)	2 (4.8)		2 (3.9)	4 (10.3)	6 (4.8)	
Preoperative VAS score, median (IQR)	6 (4–7)	7 (5–7)	7 (4–7)	0.120	7 (5–7)	7 (4–7)	7 (5–7)	0.390	7 (5–7)	7 (4–7)	7 (4–7)	0.316
Fever, *n* (%)	5 (5.4)	9 (11.1)	7 (9.7)	0.361	7 (14.6)	9 (8.6)	5 (11.9)	0.481	3 (5.9)	3 (7.7)	12 (9.7)	0.707
Preoperative laboratory tests, *n* (%)												
WBC > 10.0 × 10^9^/L	27 (29.0)	18 (22.2)	21 (29.2)	0.543	10 (20.8)	21 (20.0)	10 (23.8)	0.887	8 (15.7)	12 (30.8)	36 (29.0)	0.042
Neutrophils > 70%	29 (31.2)	28 (34.6)	25 (34.7)	0.866	16 (33.3)	33 (31.4)	15 (35.7)	0.213	17 (33.3)	21 (53.8)	55 (44.4)	0.046
CRP > 5 mg/L	26 (28.0)	31 (38.3)	26 (36.1)	0.248	20 (41.6)	34 (32.4)	15 (35.7)	0.491	18 (35.3)	19 (48.7)	55 (44.4)	0.187
Fecalith appendicitis, *n* (%)	41 (44.1)	46 (56.8)	43 (59.7)	0.096	24 (50.0)	56 (53.3)	18 (42.9)	0.423	21 (41.2)	18 (46.2)	53 (42.7)	0.932
Procedure time, min, mean ± SD	34.1 ± 7.6	31.0 ± 11.2	28.4 ± 7.3	<0.001	28.8 ± 8.5	26.7 ± 7.7	25.6 ± 3.7	0.004	23.1 ± 5.8	20.4 ± 5.4	20.2 ± 4.5	0.003
Appendiceal stent placement, *n* (%)	11 (11.8)	4 (4.9)	4 (5.6)	0.192	13 (27.1)	43 (41.0)	27 (64.3)	<0.001	28 (54.9)	16 (41.0)	47 (37.9)	0.022
Technical success rate, *n* (%)	86 (92.3)	77 (95.1)	70 (97.2)	0.604	44 (91.7)	102 (97.1)	41 (97.6)	0.216	50 (98.0)	38 (97.4)	100 (80.6)	0.558
Conversion to surgery, *n* (%)	3 (3.2)	3 (3.7)	4 (5.6)	0.727	1 (2.1)	3 (2.9)	0 (0.0)	0.561	1 (2.0)	1 (2.6)	2 (1.6)	0.823
Intraoperative complications, *n* (%)	3 (3.2)	1 (1.2)	0 (0.0)	0.191	1 (2.1)	0 (0.0)	1 (2.4)	0.652	1 (2.0)	1 (2.6)	1 (0.8)	0.618
Perforation	3 (3.2)	1 (1.2)	0 (0.0)		1 (2.1)	0 (0.0)	1 (2.4)		1 (2.0)	1 (2.6)	0 (0.0)	
Hemorrhage	3 (3.2)	0 (0.0)	0 (0.0)		1 (2.1)	0 (0.0)	0 (0.0)		0 (0.0)	1 (2.6)	1 (0.8)	
Postoperative complication, *n* (%)												
Delayed perforation	2 (2.2)	0 (0.0)	0 (0.0)	0.337	1 (2.1)	2 (1.9)	0 (0.0)	0.726	0 (0.0)	0 (0.0)	0 (0.0)	–
Postoperative VAS score at 6 h, median (IQR)	2 (0–2)	1 (0–3)	1 (0–2)	0.021	2 (0–2)	0 (0–2)	0 (0–2)	0.039	1 (0–2)	0 (0–2)	0 (0–2)	0.038
Postoperative length of stay, days, mean ± SD	2.2 ± 2.1	1.7 ± 1.6	1.3 ± 1.0	<0.001	2.1 ± 1.6	1.8 ± 1.7	1.3 ± 1.3	0.017	2.1 ± 1.0	2.0 ± 1.0	1.8 ± 0.9	0.017
Postoperative laboratory tests, *n* (%)	*n *= 45	*n *= 30	*n *= 39		*n *= 32	*n *= 78	*n *= 42		*n *= 44	*n *= 26	*n *= 100	
WBC >10.0 × 109/L	9 (20.0)	3 (10.0)	2 (5.1)	0.081	9 (28.1)	8 (10.3)	4 (9.5)	0.027	5 (9.8)	3 (7.7)	17 (13.7)	0.028
Neutrophils >70%	14 (31.1)	3 (10.0)	2 (5.1)	0.008	14 (43.8)	12 (15.4)	6 (14.2)	<0.001	15 (29.4)	5 (12.8)	17 (13.7)	0.007
CRP >5 mg/L	10 (22.2)	2 (6.7)	2 (5.1)	0.042	14 (43.8)	9 (11.5)	4 (9.5)	0.003	19 (37.3)	8 (20.5)	32 (25.8)	0.004
Recurrent appendicitis, *n* (%)	24 (25.8)	18 (22.2)	10 (13.9)	0.035	9 (18.8)	12 (11.4)	1 (2.4)	0.008	11 (21.6)	6 (15.4)	1 (0.8)	<0.001
Time to recurrence, months, median (IQR)	2.00 (1.00–12.00)	3.00 (2.00–5.50)	2.00 (1.00–3.00)	0.047	18.00 (7.00–34.00)	6.00 (3.00–12.00)	6.00 (6.00–6.00)	0.002	19.00 (10.00–25.00)	14.00 (10.00–19.25)	25.00 (25.00–25.00)	0.009
Clinical success rate, *n* (%)	76 (81.7)	71 (87.7)	66 (91.7)	0.151	41 (85.4)	97 (92.4)	40 (95.2)	0.041	44 (86.3)	36 (92.3)	117 (94.4)	0.041

ERCP, endoscopic retrograde cholangiopancreatography; SD, standard deviation; BMI, body mass index; VAS, visual analog scale; IQR, interquartile range; WBC, white blood cell count; CRP, C-reactive protein.

The technical success rate showed a significant upward trend with the accumulation of clinical practice (Fig. 4B). During the learning phase, the success rates for the novice group and the non-ERCP experience group were 93.5% and 91.7%, respectively, significantly lower than the 98.0% observed in the ERCP experience group, indicating that prior expertise in ERCP skills, including guidewire, catheterization, contrast imaging, and stone retrieval techniques, greatly facilitated early-stage ERAT learning. In the plateau phase, the success rate improved to 95.1%, 97.1%, and 97.4% in the novice group, the non-ERCP experience group, and the ERCP experience group, respectively, reflecting advancements in both procedural skills and techniques, particularly in the two groups without ERCP experience (the novice group and the non-ERCP experience group). In terms of proficient phase, the success rate reached 97.2%, 97.6%, and 100.0% in the novice group, the non-ERCP experience group, and the ERCP experience group, respectively, indicating that all groups had achieved sufficient expertise.

Postoperative pain score by VAS exhibited a statistically significant reduction across the three phases in all of the groups. The median score decreased from 2 (0–2), 2 (0–2), and 1 (0–2) in the learning phase to 1 (0–3), 0 (0–2), and 0 (0–2) in the plateau phase and to 1 (0–2), 0 (0–2), and 0 (0–2) in the proficiency phase (*P *= 0.021, 0.039, and 0.038). These results highlight enhanced technical proficiency and quick pain relief. Similarly, the length of hospital stay decreased significantly across the different phases, namely from 2.2 days, 2.1 days, and 2.1 days in the learning phase to 1.3 days, 1.3 days, and 1.8 days in the proficiency phase (*P *< 0.001, *P *= 0.017, and *P *= 0.017). A significant reduction in recurrence was observed in the three groups, decreasing from 25.8%, 18.8%, and 21.6% in the learning phase to 13.9%, 2.4%, and 0.8% in the proficiency phase (*P *= 0.035, 0.008, and < 0.001) (Fig. 4C). The clinical success rate in each group also showed a significant improvement with the accumulation of ERAT experience (Fig. 4D).

## Discussion

ERAT has emerged as an innovative, minimally invasive treatment for appendicitis, offering distinct advantages such as organ preservation and faster recovery [11, 12]. Despite its benefits, ERAT is predominantly performed in China, with limited adoption internationally [13, 14]. However, alongside its growing influence, international efforts are also being made, with occasional publications emerging [4]. Additionally, foreign physicians have begun coming to China to learn ERAT techniques. In the present study, we assessed the learning curve for ERAT using CUSUM analysis, stratified based on different experience levels in therapeutic endoscopy and ERCP, so as to facilitate the global application of this technique.

**Figure 5:**
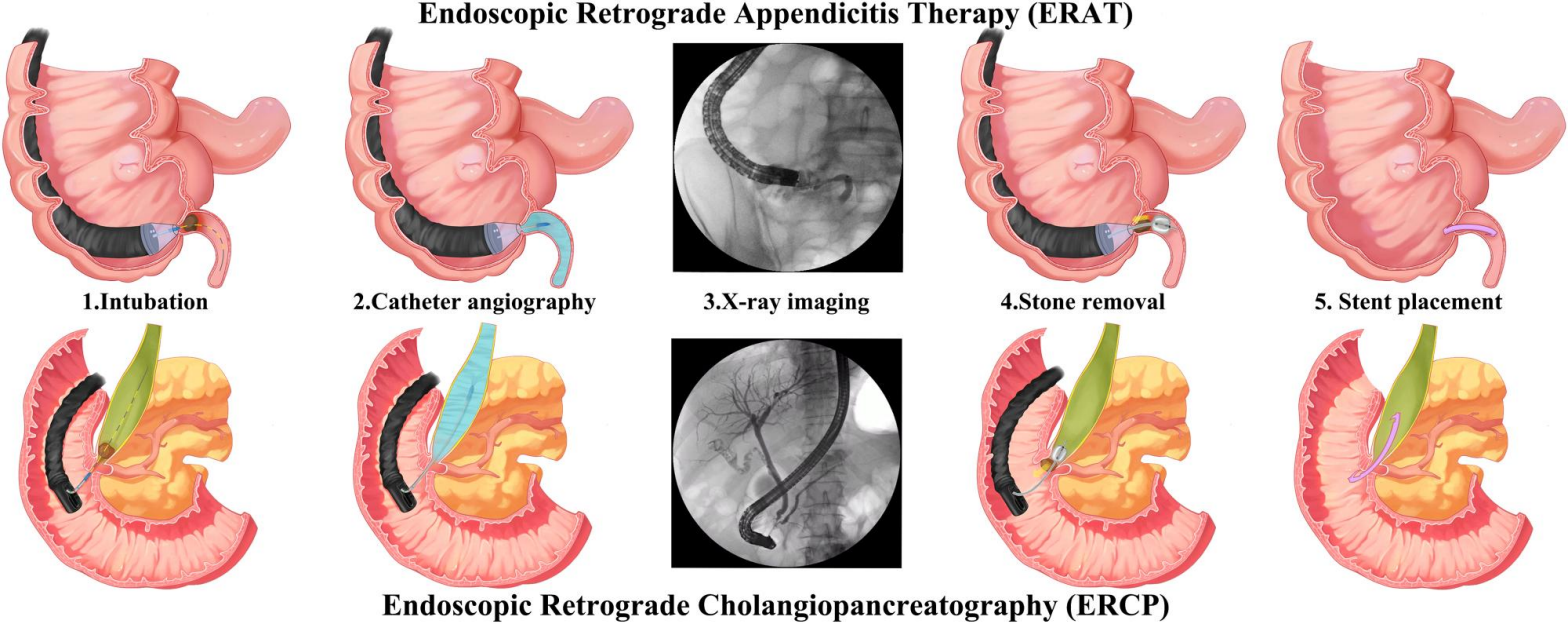
The similarities between ERAT and ERCP techniques (including intubation, catheter angiography, X-ray imaging, stone removal, and stent placement).

This study, conducted at three ERAT referral centers in China, represents the first report to characterize the ERAT learning curve. We found that trainees with sufficient endoscopic experience can master ERAT with fewer than 20 cases, proving that this technique is relatively simple and easy to master for most endoscopists. Compared with previous studies on the learning curves of advanced therapeutic endoscopic procedures such as ERCP and endoscopic submucosal dissection—which generally require a higher case volume and mentored training—the entry threshold and proficiency attainment for ERAT are relatively lower. This suggests that ERAT has good potential for dissemination in centers equipped with established endoscopy platforms [15–17]. Since ERAT and ERCP share technical similarities, such as intubation, fluoroscopic guidance, stone removal, and stent placement, it is reasonable that an ERCP-experienced trainee could acquire proficiency in ERAT more rapidly (Fig. 5 illustrates the similarities between ERAT and ERCP techniques). Our findings also corroborate this assumption; namely, the ERCP-experienced trainees required 30 cases to achieve proficiency, whereas the non-ERCP experience trainees required at least 50 cases. The structured training program developed by The First Affiliated Hospital of Zhengzhou University, which includes the acquisition of basic endoscopic skills, procedural observation, and scheduled hands-on training, has been instrumental in enabling endoscopists to implement ERAT in China [18]. Our experience in ERAT learning is meaning for all the trainees with different endoscopic experience levels worldwide. As a representative of proficiency, procedure time was significantly shorter for ERCP-competent trainees compared with novice endoscopists and non-ERCP endoscopic experts. Even after reaching proficiency, the novice and non-ERCP expert trainees had longer procedure time, underscoring the importance of ERCP skills for the application of ERAT.

Beyond procedure time, we further assessed improvements in the “quality” of the learning curve using technical and clinical success rates. With case accumulation, both technical and clinical success rates continuously improved across all three trainee groups. At the final phase of proficiency, technical success rates reached 97.2%, 97.6%, and 100%, respectively, confirming that ERAT is an easily mastered techniques. The cases of technical failure were mainly due to failed cannulation, highlighting the importance of ERCP experience. Clinical success rates reached 91.7%, 95.2%, and 94.4%, respectively. We believe this is related to novices lacking sufficient contrast imaging experience, making it difficult to determine whether appendicoliths were thoroughly cleared. Additionally, fewer stents were placed in novice group may also have played a role. Adverse events were generally infrequent; although not included in the CUSUM analysis, this finding aligns with previous reports noting the low risk of serious complications with ERAT [19–21].

In terms of comparison with existing treatment strategies, ERAT, laparoscopic appendectomy, and conservative antibiotic management should be evaluated within the same clinical decision-making framework. First, compared to laparoscopic appendectomy, ERAT offers theoretical advantages in organ preservation, reduced postoperative pain, and accelerated recovery, and may lower the risk of adhesion-related postoperative complications, presenting potential value in fertility preservation for women of reproductive age [7, 11, 22]. Second, regarding conservative antibiotic management, previous randomized controlled trials (such as APPAC and CODA) suggest that antibiotics are not inferior to surgery for short-term health outcomes [23, 24]. However, the cumulative risk of initial treatment failure or late recurrence is significant, especially in the presence of appendicoliths, where failure rates rise markedly [25]. In this context, ERAT, by allowing endoscopic visualization for appendicolith removal, may offer an organ-preserving pathway with risk re-stratification for “high-risk conservative treatment populations” (such as those with appendicoliths), potentially reducing recurrence and re-intervention rates compared with pharmacologic therapy alone.

The follow-up results of this study provide supporting evidence for the above comparisons: over a median follow-up of 26 months, recurrence rates of appendicitis decreased overall as proficiency was achieved in all three patient groups. Specifically, the recurrence rate was 8.4% in the ERCP experience group, lower than 20.7% in novices and 11.3% in the non-ERCP experience group. We hypothesize that this difference may be related to the adequacy of appendicolith removal and stent placement strategies; the two groups with more advanced endoscopic skills had higher stent placement rates (42.6% and 42.5% vs 8.1%), which may have contributed to reduced recurrence. However, the placement of stents is not without costs; potential risks such as migration, obstruction, or mucosal irritation must be balanced with larger sample sizes and standardized indications. Prospective studies are needed to clarify the scenarios in which stent placement offers maximum benefit (e.g. in patients with appendiceal tortuosity, ostial stenosis, or high risk of residual appendicoliths).

Based on our preliminary results, we recommend adopting different ERAT training approaches for endoscopists with varying levels of experience in the future. For those without ERCP experience and for beginners, it is advisable to provide more comprehensive theoretical instruction, conduct guidewire/catheter manipulation practice on simulators, and increase the number of supervised cases prior to independent procedures, ensuring adequate mastery of fundamental endoscopic skills and intubation techniques. For trainees with ERCP experience, fewer supervised cases are necessary, with the emphasis placed on understanding the anatomical variations of the appendiceal orifice and the retrieval of fecaliths.

As the first multicenter study on the ERAT learning curve, this study highlights several important factors that need to be addressed in the global promotion of ERAT and holds significant value for the further dissemination of this technique. While the findings emphasize the role of ERCP experience in ERAT skill acquisition, they also underscore the feasibility of training novice or non-ERCP experience endoscopists with adequate structured programs.

This study has several limitations. It was retrospective in design and therefore subject to potential selection bias. The sample size was relatively small, and the lack of stratification by appendicitis severity may have affected procedure times and outcomes. Precise data on the size and number of fecaliths were also unavailable, which may have influenced procedural complexity. In addition, the results are constrained by operator dependence and a relatively short follow-up period. Despite these limitations, the study provides novel insights into ERAT learning curves and offers guidance for optimizing training strategies aimed at global dissemination.

## Conclusions

ERAT is a reliable, minimally invasive treatment for acute appendicitis that provides significant clinical benefits. Endoscopists with prior ERCP experience achieve proficiency more rapidly and demonstrate higher technical success with low complication rates. Structured training programs tailored to different levels of endoscopic experience are essential for safe and effective global adoption. The learning curve characterized in this study offers a valuable framework for guiding ERAT implementation worldwide. We hope that sharing this experience will help accelerate ERAT’s global acceptance and contribute to improved patient outcomes.

## Acknowledgements

Our abstract was presented as an ePoster at 2025 ESGE Days. DOI: 10.1055/s-0045–1805811.

## Authors’ contributions

D.L. and B.L. were responsible for the study design and conceptualization. J.Fan, H.Y., M.Shi, J.Feng, N.S., and H.L. collected and acquired data. J.Z. and M.S. analyzed and interpreted data. J.Z., M.Shi, S.U., A.M.G., M.S.I., and D.L. were responsible for drafting and critical revision. D.L. and B.L. were responsible for the supervision and project oversight. All authors have read and approved the final version of the manuscript.
